# A novel risk stratification model based on tumor size and multifocality to predict recurrence in pediatric PTC: comparison with adult PTC

**DOI:** 10.3389/fendo.2023.1298036

**Published:** 2024-01-11

**Authors:** Rui Du, Ying Zhang, Jiedong Kou, Jingting Li, Chengqiu Sui, Daqi Zhang, Yantao Fu, Le Zhou, Qingfeng Fu, Fang Li, Gianlorenzo Dionigi, Nan Liang, Hui Sun

**Affiliations:** ^1^Division of Thyroid Surgery, The China-Japan Union Hospital of Jilin University, Jilin Provincial Key Laboratory of Surgical Translational Medicine, Jilin Provincial Precision Medicine Laboratory of Molecular Biology and Translational Medicine on Differentiated Thyroid Cancer, Changchun, China; ^2^Department of Laboratory Medicine, Zhongnan Hospital, Wuhan University, Wuhan, China; ^3^Department of Pathophysiology and Transplantation, University of Milan, Milan, Italy; ^4^Division of Surgery, Istituto Auxologico Italiano Instituto di Ricovero e Cura a Carattere Scientifico (IRCCS), Milan, Italy

**Keywords:** papillary thyroid cancer, recurrence, pediatric, children, adults, risk factors

## Abstract

**Background:**

Pediatric papillary thyroid cancer presents with a more advanced stage of disease than adult PTC; and it is more likely to be aggresive and distant metastases, although the survival rate is high.

**Methods:**

A retrospective observational study was performed in children and adults with PTC. Fisher’s exact, chi-square, and rank-sum tests were used to examine the differences. Univariate and multivariate Cox regression analyses were applied to determine the possible risk factors for prognosis. A Kaplan-Meier curve analysis was performed to investigate the relationship between the clinicopathological characteristics and recurrence rate.

**Results:**

The study involved 156 children and 1,244 adults with PTC. Compared to the group without recurrence, proportions of tumors measuring > 1 cm (48.3% *vs*. 90.9%) and multifocality (30.3% *vs*. 63.6%) were higher, N1b stage occurred more frequently (33.8% *vs*. 100%). However, among adult PTC patients, those with recurrence were older (76.1% *vs*. 59.4%) than those without recurrence. Risk factors for pediatric PTC recurrence included tumor size and multifocality. However, in adult PTC, the risk factor was LLNM. The newly constructed Stratification.N showed better performance, as illustrated by the fact that patients who were classified into Stratification.N 3 showed an obviously poorer prognosis (*P*=0.01 and *P*=0.00062), especially in those aged >14 years (*P*=0.0052).

**Conclusion:**

Compared with adult PTC, pediatric PTC showed unique characteristics in terms of clinical pathology and recurrence. Tumor size and multifocality were strong risk factors for pediatric PTC. Accordingly, the novel proposed risk stratification method could effectively predict the recurrence of pediatric PTC.

## Highlights

- This study not only analyzed the pathological characteristics of pediatric PTC, but also analyzed the risk factors affecting the metastasis of central cervical lymph nodes, lateral cervical lymph nodes and recurrence in children. In pediatric PTC, younger age, male, larger tumor size, multifocality are more likely to metastasise to the cervical lymph nodes. A tumor size of >1 cm and multifocality are independent risk factors for the recurrence of pediatric PTC, whereas the independent risk factor for adult recurrence is only LLNM.
- Through direct comparison with adult PTC, this study found the differences of pathological characteristics and recurrence risk factor between pediatric PTC and adult PTC.
- A new risk stratification method for recurrence was developed, which can effectively predict the prognosis of pediatric PTC. The novel children’s RRS method based on tumor size and multifocality performed well in predicting pediatric PTC recurrence. This new method provides a quick and efficient tool for clinicians to evaluate the recurrence risk of patients with pediatric PTC in clinical practice and facilitate decision-making regarding appropriate treatment strategies.

## 1 Introduction

Thyroid cancer in childhood is rare, accounting for 1.5% to 3.0% of all childhood cancers. However, it has been reported that the incidence is increasing and is currently the second most common malignancy (1–3) Papillary thyroid cancer (PTC) is the most common thyroid cancer in both pediatric and adult patients. Compared with adult PTC, pediatric PTC has a higher rate of neck lymph node metastases and recurrence rates and has unique pathological features (2, 4, 5). Several studies (6–8) have shown that the biological behavior of pediatric PTC is more aggressive, and its prognosis appears to be worse. Current guidelines rarely differentiate between children and adults when stratifying the risk of recurrence of PTC (9). The aim of our study was to describe pediatric PTC and propose a framework for recurrence risk stratification (RRS) based on a comparative cohort of adults.

## 2 Materials and methods

### 2.1 Time frame, setting, and study design

A retrospective observational study was performed from June 2008 to December 2021. Children and adults who underwent surgical treatment for thyroid nodules at the Department of Thyroid Surgery, China-Japan Union Hospital, Jilin University were selected. A total of 156 children and young adults (≤20 years old) were selected. Accordingly, 1,244 adults with PTC aged > 20 years formed the adult cohort. This work has been reported in line with the STROCSS criteria (10).

### 2.2 Ethics

This study was approved by the Ethics Review Board of China-Japan Union Hospital (No. 20220804012). Informed consent was obtained from the participants or their guardians. The study was conducted in accordance with the Declaration of Helsinki.

### 2.3 Inclusion and exclusion criteria

The inclusion criteria were as follows: (1) first thyroid surgery was performed in our department; (2) postoperative paraffin pathology showed a diagnosis of PTC; (3) complete follow-up data up to 2021.

The exclusion criteria were as follows: (1) patients with other malignancies; (2) lack of required relevant information.

### 2.4 Data collection

Data on patients’ basic demographic information (sex, age, body mass index, and family history), clinical presentation (tumor size, extrathyroidal extension [ETE], multifocality, localization, lymph node metastases, Hashimoto’s thyroiditis [HT], and nodal goiter [NG]), surgical data (type of surgery, the extent of lymph node dissection, and complications), postoperative paraffin pathology, and postoperative radioactive iodine administration (RAI) were collected. Recurrence rates were obtained from the hospital and outpatient clinic medical records database. Tumor stage and RRS were determined by experienced thyroid surgery physicians.

### 2.5 Surgical treatment

Pediatric patients with PTC were treated based on the American Thyroid Association (ATA) management guidelines. According to the ATA guidelines, bilateral total thyroidectomy is recommended for most children because of the increased incidence of bilaterality and multifocal disease in this population. However, in the study patients, lobectomy was only performed when the disease was obviously limited, such as in the case of an intrathyroidal lesion or when no bilaterality was noted on preoperative assessment. Prophylactic central compartment node dissection was regularly performed in all pediatric patients.

### 2.6 Postoperative management and follow-up

The follow-up period included the time between the date of surgery and the last clinical follow-up. Thyroid function tests (including serum T3, T4 and thyroid stimulating hormone [TSH], Tg and TgAbs), neck ultrasound, and computed tomography were performed in all included patients. Preoperative fine-needle aspiration cytology and postoperative pathological analyses were performed in the patients. All pediatric and adult patients were managed postoperatively according to the guidelines of ATA. RAI ablation was performed 4 to 6 weeks postoperatively, with dosing according to the guidelines of ATA. Whole body scans (WBS) were performed 5 to 7 days after RAI ablation in patients undergoing total thyroidectomy (TT). Thyroglobulin (Tg) and antithyroglobulin antibody (TgAb) concentrations were determined after TSH stimulation by T4 deprivation or recombinant human TSH before RAI ablation. All patients received L-thyroxine at suppressive doses and underwent physical examination, thyroid function tests, determination of Tg and TgAb concentrations, and neck ultrasound every 3 to 6 months and annually thereafter. In patients who showed signs of recurrence or distant metastasis at routine follow-up, additional imaging techniques, such as computed tomography, positron emission tomography, and/or RAI WBS, were used to determine the location and extent of the suspected recurrence.

### 2.7 Definitions

The tumor stages were defined according to the Eighth Edition of the American Joint Committee on Cancer. Recurrence was confirmed by structure recurrence, which performed for patients with biopsy-proven persistent or recurrent disease for central neck nodes more than 8 mm and lateral neck nodes more than 10 mm in the smallest dimension that can be localized on anatomic imaging after central and/or lateral neck dissection 1 year later. The RRS was determined based on the ATA’s 2015 management guidelines for pediatric and adult thyroid cancer. Individuals with a BMI greater than 24 but less than 29 were defined as overweight, and those with a BMI greater than 29 were defined as obese. The receiver operating characteristic (ROC) curve analysis was used to determine the optimal cut-off points for patients whose age was associated with the prediction of structural persistent/recurrent disease.

Multifocal papillary thyroid carcinoma (PTC) is defined as the presence of two or more clinically relevant nodules, either unilaterally or bilaterally within the thyroid gland, indicating the existence of two or more cancerous foci. The 2009 ATA Initial Risk Stratification System is recommended for DTC patients treated with thyroidectomy, based on its utility in predicting risk of disease recurrence and/or persistence. (Strong recommendation, Moderate-quality evidence). Low-risk patients were defined as having intrathyroidal DTC with no evidence of extrathyroidal extension, vascular invasion, or metastases. High risk patients had gross extrathyroidal extension, incomplete tumor resection, distant metastases, or inappropriate postoperative serum Tg values.

### 2.8 Statistical analysis

SPSS (version 22.0) software was used to analyze the data in the present study. Continuous variables were described as medians with interquartile ranges, and categorical variables were described as numbers with percentages. Fisher’s exact test, chi-square test, and rank sum test were used to determine the difference between the two groups. Univariate and multivariate Cox regression analyses were used to identify the risk factor of prognosis. The Kaplan–Meier curve analysis and ROC curve analysis were used to explore the relationship between clinical pathological features and tumor recurrence. Risk stratification was performed according to the factors related to disease-free survival. Hazard ratios [HR] with 95% confidence intervals [CI] were also estimated. Further, *P*<0.05, which is two-sided, was considered statistically significant.

## 3 Results

### 3.1 Demographic data

A total of 156 children and young adults (≤20 years old) were selected. Accordingly, 1,244 adult patients with PTC aged > 20 years formed the adult cohort. Demographic characteristics are summarized in **Table S1**. The median follow-up time for the child and adult cohorts was 142 weeks (range, 81.4~287.9 weeks) and 258.86 weeks (range, 57.29~606.71 weeks), respectively.

### 3.2 Unique clinical pathological features of pediatric papillary thyroid cancer

In this study, 156 patients with pediatric PTC were enrolled during the 2008–2021 period. At the 142-week follow-up (range, 81.4~287.9-week follow-up), 7.1% of them experienced a recurrence (**Table 1**). To explore the differential clinical pathological features within increasing diagnostic ages, patients with pediatric PTC were divided into the following two groups: those under 14 years in the younger group and those above 14 years in the older group. As shown in **Table S2**, the proportion of those with a tumor size > 1 cm was higher in the younger group (under 14 years) compared with the older group (73.1% vs. 46.9%, χ^2^ = 0.015, *P=*0.028). Further, the proportion of central cervical lymph node metastasis (CLNM) was higher (92.3% vs. 67.7%, χ^2^ = 6.483, *P=*0.011), with a higher T4 stage proportion (15.4% vs. 0.8%, Z=-2.956, *P=*0.003) in the younger pediatric PTC group. In addition, the recurrence rate was slightly higher in the younger pediatric PTC group than in the older group, although no obvious difference was observed (18.2% vs 17.2%, P=0.6). This suggests that the biological behavior of pediatric PTC in younger patients is more aggressive. In addition, the possible factors of CLNM were further analyzed. As illustrated in **Table S3**, a larger tumor size (63.4% vs. 20.5%, χ^2^ = 23.312, *P*<0.001), higher T stage (4.5% vs. 0%, Z=-3.286, *P*=0.001), higher N stage (50.9% vs. 6.8%, Z=-9.287, *P*<0.001), and higher RRS (44.6% vs. 6.8%, χ^2^ = 114.561, *P*<0.001) might be associated with CLNM. Similarly, except for the above factors, lateral cervical lymph node metastasis (LLNM) was still associated with multifocality (47.5% vs. 16.7%, χ^2^ = 3.875, *P*=0.049) (**Table S4**). The above data indicated the unique biological behaviors of pediatric PTC.

**Table 1:** Baseline characteristics of papillary thyroid cancer in children (N=156).

Characteristics	Children N (%)	Characteristics	Children N (%)
**Number**	156	Yes	50 (32.05)
**Sex**		**NG (included**	
Female	123 (78.85)	No	67 (42.95)
Male	33 (21.15)	Yes	89 (57.05)
**Age**		**T stage**	
≤14	26 (16.67)	T1	127 (81.41)
>14	130 (83.33)	T2	19 (12.18)
**BMI**		T3	5 (3.21)
Normal	114 (73.08)	T4	5 (3.21)
Over-weight	17 (10.90)	**N stage**	
Obese	25 (16.03)	N0	41 (26.28)
**Family history**		N1a	55 (35.26)
No	143 (91.67)	N1b	60 (38.46)
Yes	13 (8.33)	**M stage**	
**Primary tumor size**		M0	153 (98.08)
≤1 cm	76 (48.72)	M1	3 (1.92)
>1 cm	80 (51.28)	**Treatment**	
**Extrathyroidal extension**		TT	81 (51.92)
No	117 (75.00)	<TT	75 (48.08)
Yes	39 (25.00)	**Outcome**	
**Multifocality**		Non-recurrence	145 (92.95)
No	105 (67.31)	Recurrence	11 (7.05)
Yes	51 (32.69)	**RAI**	
**Location**		No	89 (57.05)
Unilateral	127 (81.41)	Yes	67 (42.95)
Bilateral	29 (18.59)	**Complications**	
**CLNM**		No	145 (92.95)
No	44 (28.21)	Yes	11 (7.05)
Yes	112 (71.79)	**RRS**	
**LLNM**		Low	43 (27.56)
No	12 (16.90)	Intermediate	60 (38.46)
Yes	59 (83.1)	High	53 (33.97)
**HT**		**Follow time** (weeks)	142 [81.4,287.9]
No	106 (67.95)		

BMI, Body Mass Index; HT, Hashimoto’s thyroiditis; NG (included), Nodular Goiter; CND, Central Cervical Lymph Node Dissection; RAI, Radioactive iodine; RRS, Recurrence risk stratification.

### 3.3 Possible relative pathological features of recurrence differed between pediatric PTC and adult PTC

To explore the risk factors of recurrence, the patients were divided into the recurrent and non-recurrent groups. As shown in **Table 2**, individuals experiencing recurrence in the pediatric PTC group exhibited larger tumor diameters (90.9% vs. 48.3%, χ^2^ = 7.438, *P*=0.006), more multifocality (63.6% vs. 30.3%, *P*=0.03), a more bilateral shape (45.5% vs. 16.6%, *P*=0.032), a higher prevalence of lateral cervical lymph node metastasis (50% vs. 13.1%, *P*=0.019), and an elevated RRS (90.9% vs. 29.7%, Z=-3.757, *P*<0.001) compared with those who did not experience any recurrence. As this study aimed to investigate the different risk factors of recurrence between pediatric and adult PTC, another adult PTC cohort was formed (**Table S1**). As illustrated in **Table 2**, in adult PTC, factors, such as a older age (59.4% *vs*. 40.6%, χ^2^ = 4.718, *P*=0.03), CLNM (68.8% vs. 43.4%, χ^2^ = 8.129, *P*=0.004), and a higher LLNM (88.9% vs. 47.7%, χ^2^ = 11.768, *P*<0.001) were associated with tumor recurrence. These results suggest that the relative factors of recurrence between pediatric and adult PTC are not the same.

**Table 2:** Clinicopathological characteristics of papillary thyroid cancer in recurrent and non-recurrent groups of children and adults.

Characteristics	Children				Adult				Characteristics	Children				Adult			
	non-Rec N (%)	Recurrence N (%)	Z/χ2	*P* value	non-Rec N (%)	Recurrence N (%)	Z/χ2	*P* value		non-Rec N (%)	Recurrence N (%)	Z/χ2	*P* value	non-Rec N (%)	Recurrence N (%)	Z/χ2	*P* value
**Total**	145(92.9)	11(7.1%)			1212(97.4)	32(2.6)	Fisher	0.006**	**Treatment**			8.697	0.003**			4.253	0.039*
**Sex**				0.422			1.402	0.236	<TT	80(55.2)	1(9.1)			564(46.5)	9(28,1)		
Female	115(79.3)	8(72.7)	Fisher		1006(83.0)	24(75.0)			TT	65(44.8)	10(90.9)			648(53.5)	23(71.9)		
Male	30(20.7)	3(27.3)			206(17.0)	8(25.0)			**CLNM**				0.023*			8.129	0.004**
**Age**				0.6					No	44(30.3)	0(0)	Fisher		686(56.6)	10(31.3)		
≤14	25(17.2)	2(18.2)	Fisher		—	—			Yes	101(69.7)	11(100)			526(43.4)	22(68.8)		
>14	120(82.8)	9(81.8)			—	—	4.718	0.03*	**LLNM.cat**				0.136			11.768	<0.001***
<35	—	—			290(23.9)	13(40.6)			No	12(19.7)	0(0)	Fisher		252(52.3)	2(11.1)		
≥35	—	—			922(76.1)	19(59.4)			Yes	49(80.3)	10(100)			230(47.7)	16(88.9)		
**BMI**				0.278			1.996	0.369	**LLNM**				0.019*			13.284	0.001**
Normal	105(72.4)	9(81.8)			649(54.6)	17(54.8)			None	12(19.7)	0(0)			252(52.3)	2(11.1)		
Overweight	25(17.2)	0(0)	Fisher		426(35.9)	13(41.9)			Unilateral	41(67.2)	5(50)	Fisher		199(41.3)	14(77.8)		
Obese	15(10.3)	2(18.2)			113(9.5)	1(3.2)			Bilateral	8(13.1)	5(50)			31(6.4)	2(11.1)		
**Family history**				0.371				0.667	**T stage**			-2.571	0.01*			-2.686	0.007**
No	132(91.0)	11(100)	Fisher		1167(96.3)	31(96.9)	Fisher		T1	121(83.4)	6(54.5)			1132(93.4)	26(81.3)		
Yes	13(9.0)	0(0)			45(3.7)	1(3.1)			T2	17(11.7)	2(18.2)			48(4.0)	3(9.4)		
**Tumor size**			7,438	0.006**			2.147	0.143	T3	4(2.8)	1(9.1)			14(1.2)	2(6.3)		
≤1cm	75(51.7)	1(9.1)			928(76.8)	21(65.6)			T4	3(2.1)	2(18.2)			18(1.5)	1(3.1)		
>1cm	70(48.3)	10(90.9)			281(23.2)	11(34.4)			**N stage**			-3.894	<0.001***			-3.237	0.001**
**Multifocality**				0.03*			3.266	0.071	N0	41(28.3)	0(0)			639(52.7)	10(31.3)		
No	101(69.7)	4(36.4)	Fisher		723(59.7)	14(43.8)			N1a	55(37.9)	0(0)			341(28.1)	7(21.9)		
Yes	44(30.3)	7(63.6)			489(40.3)	18(56.3)			N1b	49(33.8)	11(100)			232(19.1)	15(46.9)		
**Localization**				0.032*			0.565	0.452	**RAI**				<0.001***			9.675	0.008**
Unilateral	121(83.4)	6(54.5)	Fisher		869(71.7)	21(65.6)			No	89(61.4)	0(0)			849(70.0)	14(43.8)		
Bilateral	24(16.6)	5(45.5)			343(28.3)	11(34.4)			Yes	56(38.6)	11(100)	Fisher		322(26.6)	15(46.9)		
**ETE**				0.107			0.429	0.513	Recommended	—	—			41(3.4)	3(9.4)		
No	111(76.6)	6(54.5)	Fisher		860(71.0)	21(65.6)			**Complications**				0.175			2.609	0.106
Yes	34(23.4)	5(45.5)			352(29.0)	11(34.4)			No	136(93.8)	9(81.8)	Fisher		942(77.7)	21(65.6)		
**HT**				0.508			2.094	0.148	Yes	9(6.2)	2(18.2)			270(22.3)	11(34.4)		
No	98(67.6)	8(72.7)	Fisher		928(76.6)	28(87.5)			**RRS**			-3.757	< 0.001***			4.18	0.124
Yes	47(32.4)	3(27.3)			284(23.4)	4(12.5)			Low	43(29.7)	0(0)			672(55.4)	16(50.0)		
**NG (include)**							0.367	0.545	Intermediate	59(40.7)	1(9.1)			495(40.8)	12(37.5)		
No	62(42.8)	5(45.5)	Fisher	0.551	285(23.5)	9(28.1)			High	43(29.7)	10(90.9)			45(3.7)	4(12.5)		
Yes	83(57.2)	6(54.5)			927(76.5)	23(71.9)											

BMI, Body Mass Index; ETE, Extrathyroidal extension; HT, Hashimoto’s thyroiditis; NG (included), Nodular Goiter; CND, Central Cervical Lymph Node Dissection; LND, Lateral cervical lymph node dissection; CLNM, Central cervical lymph node metastasis; LLNM, Lateral cervical lymph node metastasis; LLNM.cat, Lateral cervical lymph node metastasis-categorical variable; RAI, Radioactive iodine; RRS,Recurrence risk stratification.*P<0.05,**P<0.01,***P<0.001.

### 3.4 Differential risk factors of recurrence between pediatric PTC and adult PTC

**Figure 1:**
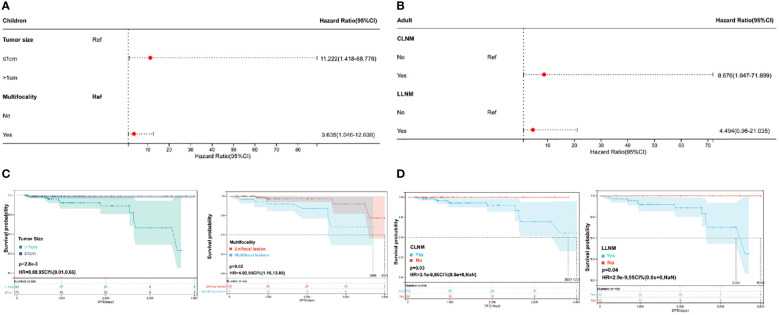
Recurrence-relative factors for children and adults. **(A)** Multivariate Cox regression analysis of pediatric papillary thyroid cancer (PTC); **(B)** multivariate Cox regression analysis of adult PTC; **(C)** Kaplan−Meier plots of tumor size and multifocality in pediatric PTC; and **(D)** Kaplan−Meier plots of CLNM and LLNM in adult PTC.

To explore the risk factors affecting recurrence in pediatric and adult PTC, a Cox regression analysis was performed (**Table 3**). According to the univariate and multivariate COX regression analyses, tumor size and multifocality were possible independent risk factors for pediatric PTC (**Figures 1A, B**). Compared to patients with a tumor size ≤ 1 cm, patients with a tumor size > 1 cm had a 11.2 times higher risk of recurrence. The risk of recurrence was 3.6 times higher in pediatric PTC patients with multifocality compared to those who had unifocal tumors. Moreover, based on the results of the multivariate analysis, Kaplan–Meier curves were plotted to explore the risk factors of pediatric PTC. As illustrated in **Figure 1C**, patients with a tumor size > 1 cm (HR =0.08, 95% CI: 0.01-0.66, *P*=0.0028) and multifocality (HR =4.02, 95% CI: 1.16–13.88, *P*=0.02) had poorer prognoses over time. The impact of other clinical and pathological characteristics was also analyzed by the Kaplan–Meier curve in pediatric PTC patients. As shown in **Figure S1**, patients with a more higher T stage had a larger risk of recurrence over time. However, regarding adult PTC, LLNM was an independent risk factor for recurrence (HR =5.231 (1.102–24.828), *P*=0.037). Furthermore, based on the clinical experience and clinical features, CLNM and LLNM were selected to plot the Kaplan–Meier curve. As shown in **Figure 1D**, patients with CLNM (HR =0.0003, 95% CI: 0.00–0, *P*=0.03) and LLNM (HR =0.0002, 95% CI: 0.00–0, *P*=0.04) had significantly poorer prognoses over time. Based on the above results, the clinical features affecting the prognosis of thyroid cancer differed between children and adults. Therefore, it is necessary to develop a new risk stratification method specifically for pediatric PTC.

**Table 3:** Univariate/Multivariate COX regression analysis for prognosis of papillary thyroid cancer in children and adults.

Characteristics		Children (Univariate)		Children (Multivariate)		Adult (Univariate)		Adult (Multivariate)	
		HR	*P*	HR	*P*	HR	*P*	HR	*P*
Age	≤14	2.18 (0.45–10.552)	0.333	—	—	0.968 (0.931–1.005)#	0.093	—	—
Sex	Female	0.443 (0.11–1.789)	0.53	—	—	0.536 (0.24–1.197)	0.128	—	—
Tumor size	>1cm	11.951 (1.519–94.016)	0.018*	11.222 (1.418-88.776)	0.022**	1.644 (0.791–3.417)	0.183	—	—
Multifocality	Yes	4.015 (1.161–13.884)	0.028*	3.635 (1.046-12.638)	0.042*	1.986 (0.987–3.999)	0.055	—	—
Location	Bilateral	2.98 (0.904–9.82)	0.073	—	—	1.335 (0.643–2.77)	0.438	—	—
ETE	Yes	2.89 (0.872–9.581)	0.083	—	—	1.917 (0.898–4.093)	0.093	—	—
CLNM	Yes	36.071 (0.135–9620.8)	0.208	—	—	3.231 (1.527–6.84)	0.002**	7.805 (0.938–64.971)	0.057
LLNM	Yes	38.443 (0.105–14044.6)	0.225	—	—	10.41 (2.384–45.465)	0.002**	5.231 (1.102–24.828)	0.037*
HT	Yes	0.976 (0.254–3.754)	0.971	—	—	0.398 (0.139–1.142)	0.087	—	—
NG (included)	Yes	1.134 (0.338–3.809)	0.839	—	—	0.842 (0.389–1.822)	0.663	—	—
T stage	T2	2.458 (0.493–12.243)	0.272	—	—	2.73 (0.826–9.022)	0.1	0.769 (0.172–3.443)	0.731
(Ref: T1)	T3	7.01 (0.784–62.71)	0.081	—	—	5.361 (1.266–22.708)	0.023*	5.258 (1.163–23.775)	0.031
	T4	13.137 (2.333–73.979)	0.003**	—	—	2.414 (0.327–17.848)	0.388	1.398 (0.181–10.795)	0.748

BMI, Body Mass Index; ETE, Extrathyroidal extension; HT, Hashimoto’s thyroiditis; NG (included), Nodular Goiter; CND, Central Cervical Lymph Node Dissection; LND, Lateral cervical lymph node dissection; CLNM, Central cervical lymph node metastasis; LLNM, Lateral cervical lymph node metastasis; LLNM.cat, Lateral cervical lymph node metastasis-categorical variable; RAI, Radioactive iodine; RRS, Recurrence risk stratification. *P< 0.05, **P<0.01. # Adult age ≤35 years group.

### 3.5 Good performance of the novel recurrence risk stratification (Stratification.N) for pediatric papillary thyroid cancer

Based on the above results, a new risk stratification method was proposed in pediatric PTC, which was named Stratification.N. Tumor size > 1 cm and multifocality were incorporated as the basis for classification. Patients with a tumor size ≤ 1 cm and single focus were classified into Stratification.N 1. Patients with a tumor size > 1 cm or those with multifocal tumors were classified into Stratification.N 2. Patients with a tumor size > 1 cm and multifocality were classified into Stratification.N 3.

**Figure 2:**
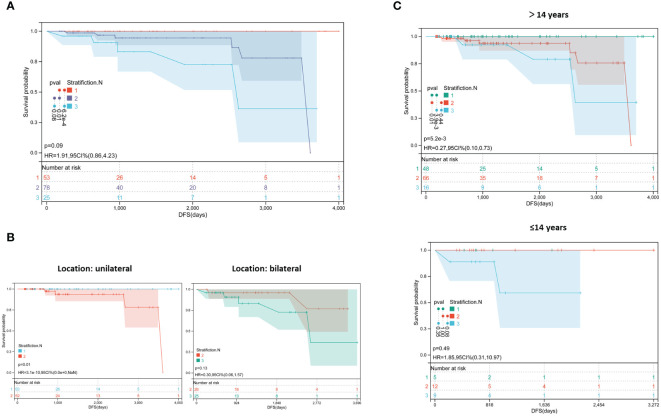
Survival analysis of the new risk stratification method. **(A)** Kaplan−Meier plots of novel recurrence risk stratifications in pediatric PTC; **(B)** Kaplan−Meier plots with different stratification in patients with unilateral and bilateral cancer; **(C)** Kaplan−Meier plots with with different stratification in patients with different age group.

Furthermore, Kaplan–Meier curves were plotted to evaluate the performance. As shown in **Figure 2A**, patients classified into Stratification.N 3 had an obviously poorer prognosis among the three groups (*P*=0.01 and *P*=0.00062). Furthermore, the patients were divided into two groups based on unilateral and bilateral thyroid cancer. As shown in **Figure 2B**, patients with unilateral thyroid cancer, who were classified into Stratification.N 2, had significantly poorer prognoses (HR =0.0003, 95% CI: 0.00–0, *P*=0.01). As shown by the Kaplan–Meier curve, patients with bilateral thyroid cancer, who had tumors located in the third layer, had a worse prognosis trend than those who had tumors located in the second layer (*P*=0.13). This may be due to the small sample size and short follow-up time. To further evaluate the efficiency of Stratification.N in different age groups, specific Kaplan–Meier curves were plotted. As shown in **Figure 2C**, among patients aged >14 years, those classified into Stratification.N 3 had a worse prognosis (HR =0.27, 95% CI: 0.1–0.73, *P*=0.0052), suggesting that Stratification.N could better predict the likelihood of recurrence of pediatric PTC. However, Stratification.N did not show a good performance in adult PTC (data not shown), possibly due to the differential risk factors (only LLNM for adult PTC). Therefore, Stratification.N might be used to predict the risk of recurrence more accurately in pediatric PTC.

## 4 Discussion

The incidence of pediatric thyroid cancer has been increasing year by year, and it has now become the second most common malignancy in children (2, 11). However, the 2015 ATA guidelines for pediatric thyroid cancer follow the same risk stratification method as those for adult thyroid cancer. Currently, there is a lack of an RRS method specifically developed based on the characteristics of pediatric thyroid ca. Therefore, it is not possible to accurately predict the likelihood of recurrence in pediatric thyroid cancer patients (12). In this study, the authors first investigated the pathological characteristics of pediatric PTC and how it differed from adult PTC and then they explored the differential risk factors for pediatric PTC and adult PTC. Finally, they proposed Stratification N. to predict the recurrence of pediatric PTC more accurately.

This study first described the unique biological behaviors of pediatric PTC. Despite the 2015 American Thyroid Association (ATA) Pediatric Thyroid Cancer Guidelines recommending a research age limit of 18 years and below, our study has chosen to extend the age criteria to 20 years and below. This decision stems from a synthesis of literature findings and practical work experience (13–15), driven by the desire to juxtapose the characteristics of pediatric thyroid cancer across diverse age groups. Patients with pediatric PTC under the age of 14 years exhibited larger tumor diameters and a higher rate of CLNM. Tumor size, and multifocality were associated with CLNM. In the retrospective research by Chen, among the 52 cases, the proportion of local invasion in the pre-school group was found to be higher than that in the school-age group (16). As shown by Huang’s research (17), patients with positive preoperative TPOAb (*P*=0.047) and TgAb (*P*=0.047) had a lower recurrence rate in the younger group. However, this study found no significant difference in the proportion of bilateral cancer and concurrent thyroiditis in pediatric patients with PTC compared with adult patients with PTC. Compared with adult PTC, pediatric PTC exhibited differences in pathophysiology, clinical presentation, and long-term outcomes.

In recent years, an increasing number of scholars have proposed that the clinical pathological features of pediatric PTC and adult PTC are not entirely consistent. Gui’s research found that a younger age at diagnosis, positive preoperative TSH, a maximum tumor size >2 cm, lateral LNM, number of LNMs, N staging, and RRS were related to a poor prognosis in patients with PTC (*P*<0.05) (18). Wang explored 15 years of data from their center, and they found that younger age and ETE were significant dependent factors of post-operative recurrence (19). Guo’s study (20) found that age, bilateral involvement, extrathyroidal invasion, concurrent thyroiditis, and multifocality were risk factors for lateral neck lymph node metastasis in pediatric PTC. This study found that a younger age, larger tumor size, and lateral neck lymph node metastasis were associated with an increased risk of CLNM. Meanwhile, a larger tumor size, multifocality, and CLNM were associated with an increased risk of lateral neck lymph node metastasis in pediatric PTC. This is consistent with the characteristics of pediatric PTC found in other studies (16, 19, 21). In addition, several studies (22–25) suggested that NTRK and RET gene fusion might play an important role in the occurrence and development of pediatric thyroid cancer. Therefore, the characteristics of pediatric PTC patients are significantly different from those of adult PTC patients.

An increasing number of studies have proposed that the prognosis of pediatric and adult PTC is also not entirely the same. As shown by Chen’s study, over half of the recurrent cases had a low T stage and low ATA risk levels at the initial diagnosis (78.3% and 51.4%) (16). Geiger, after 21 years of investigation, found that in patients with PTC, positive surgical margins, node positive disease, and a tumor size of 3 cm or more were risk factors for a lower locoregional recurrence-free survival rate (26). In this study, compared to patients with non-recurrent pediatric PTC, patients with recurrent pediatric PTC were more likely to have a tumor size larger than 1 cm, multifocality, bilateral involvement, and lateral neck lymph node metastasis. Patients with recurrent adult PTC were more likely to be over 35 years of age, and have central or lateral neck lymph node metastasis. Risk factors for recurrence in adult PTC included lateral neck lymph node metastasis and CLNM. Tumor size and multifocality were independent risk factors for recurrence in pediatric PTC patients. However, Guo’s study (20) suggested that tumor size, bilateral involvement, concurrent thyroiditis, and lateral neck lymph node metastasis might also affect recurrence in pediatric PTC patients, with concurrent thyroiditis being an independent risk factor for recurrence. In our clinical practice, we observed a higher recurrence rate in patients undergoing secondary surgeries who were initially between the ages of 10 and 16 compared to those aged between 16 and 20 at their first surgery. This observation led us to hypothesize that younger patients may exhibit a higher susceptibility to recurrence. Regrettably, our statistical findings in this study did not corroborate our clinical observations, possibly owing to the relatively short follow-up duration and the limited sample size. Some studies (17, 20) have suggested that the development of pediatric thyroid cancer is influenced by local inflammatory cells, which may promote the spread of tumor cells. This demonstrates that the clinical characteristics affecting recurrence in children are different from those affecting recurrence in adults.

Accordingly, it is necessary to conduct a differential risk assessment to better distinguish and evaluate pediatric PTC and adult PTC. In clinical practice, clinicians need a quick and convenient way to assess the risk of recurrence in patients and decide upon specific treatment options based on the results (27, 28). Therefore, it is necessary to develop an RRS method that can be easily applied in clinical settings (12, 29). Many studies (18, 30, 31) have suggested that tumor size and lymph node metastasis are important factors for predicting recurrence in pediatric PTC. In this study, based on the systemic analysis, two important clinical features—tumor size and multifocality—were included as key factors in the RRS method. The study found that this new method can clearly distinguish patients with pediatric PTC with different levels of recurrence risk. In combination with the age and location of tumor, it can better predict the recurrence risk in high-risk patients. This new method provides a quick and efficient tool for clinicians to evaluate the recurrence risk in patients with pediatric PTC in clinical practice and facilitate decision-making regarding appropriate treatment strategies.

The limitations of this study are that it was a single-center study and represented a certain limitation to the population. Additionally, the follow-up time of this study was relatively short and it needs to be further extended. Thyroid cancer has a relatively low overall mortality rate, and therefore, recurrence would be a desirable outcome when discussing the risks of the extent of surgery. Therefore, future population-based studies on recurrence are needed. Furthermore, we did not include additional treatment modalities, such as radioactive iodine therapy, which may influence the physician’s decision regarding the extent of surgery. In addition, patients with certain pathological features, including disseminated stages and a size of 40 mm or more, have limited representation, limiting the statistical power for these patients. Despite the limitations of our database, this analysis provides one of the most comprehensive views on thyroidectomy in pediatric patients in China.

## 5 Conclusion

In pediatric PTC, tumors that have a larger size and multifocality are more likely to metastasize to the cervical lymph nodes. Tumor size > 1 cm and multifocality are independent risk factors for the recurrence of pediatric PTC, whereas the independent risk factor for adult recurrence is only LLNM. This novel pediatric RRS method based on tumor size and multifocality showed a good performance in predicting pediatric PTC recurrence. It can be combined with age to further improve the accuracy. However, its performance should be validated in larger, multi-center cohorts in the future.

## Data availability statement

The raw data supporting the conclusions of this article will be made available by the authors, without undue reservation.

## Ethics statement

The studies involving humans were approved by China-Japan Union Hospital Institutional Review Board (No. 20220804012). The studies were conducted in accordance with the local legislation and institutional requirements. The participants provided their written informed consent to participate in this study.

## Author contributions

RD: Data curation, Formal analysis, Methodology, Writing – original draft, Writing – review & editing, Validation. YZ: Conceptualization, Data curation, Writing – review & editing. JK: Formal analysis. JL: Data curation, Writing – review & editing. CS: Data curation, Writing – review & editing. DZ: Data curation, Writing – review & editing. YF: Data curation, Writing – review & editing. LZ: Data curation, Writing – review & editing. QF: Data curation, Writing – review & editing. FL: Data curation, Writing – review & editing. GD: Supervision, Validation, Visualization, Writing – review & editing. NL: Conceptualization, Data curation, Formal analysis, Funding acquisition, Investigation, Methodology, Resources, Supervision, Visualization, Writing – original draft, Writing – review & editing. HS: Conceptualization, Funding acquisition, Investigation, Methodology, Project administration, Supervision, Visualization, Writing – original draft, Writing – review & editing.

## Supplementary material

Supplementary Figure 1 Recurrence-relative factors of children. Kaplan−Meier plots of the following: **(A)** Gender, **(B)** Age group, **(C)** BMI, **(D)** Family history, **(E)** ETE, **(F)** Location, **(G)** HT, **(H)** NG, **(I)** T stage, **(J)** N stage, **(K)** M stage, and **(L)** Surgery in pediatric PTC.

## References

[1] Zheng R Zhang S Zeng H Wang S Sun K Chen R. Cancer incidence and mortality in China. J Natl Cancer Center (2016) 2(1):1–9. doi: 10.1016/j.jncc.2022.02.002 PMC11256658 39035212

[2] Vaccarella S Lortet-Tieulent J Colombet M Davies L Stiller CA Schuz J. Global patterns and trends in incidence and mortality of thyroid cancer in children and adolescents: a population-based study. Lancet Diabetes Endocrinol (2021) 9(3):144–52. doi: 10.1016/S2213-8587(20)30401-0 33482107

[3] van de Berg DJ Kuijpers AMJ Engelsman AF Drukker CA van Santen HM Terwisscha van Scheltinga S. Long-term oncological outcomes of papillary thyroid cancer and follicular thyroid cancer in children: A nationwide population-based study. Front Endocrinol (Lausanne) (2022) 13:899506. doi: 10.3389/fendo.2022.899506 35600573 PMC9114695

[4] Redlich A Luster M Lorenz K Lessel L Rohrer TR Schmid KW. Age, american thyroid association risk group, and response to therapy are prognostic factors in children with differentiated thyroid cancer. J Clin Endocrinol Metab (2022) 107(1):e165–77. doi: 10.1210/clinem/dgab622 34415989

[5] Sugino K Nagahama M Kitagawa W Ohkuwa K Matsuzu K Suzuki A. Cutoff age between pediatric and adult thyroid differentiated cancer: is 18 years old appropriate? Thyroid (2022) 32(2):145–52. doi: 10.1089/thy.2021.0255 34549602

[6] Pasqual E Schonfeld S Morton LM Villoing D Lee C Berrington de Gonzalez A. Association between radioactive iodine treatment for pediatric and young adulthood differentiated thyroid cancer and risk of second primary Malignancies. J Clin Oncol (2022) 40(13):1439–49. doi: 10.1200/JCO.21.01841 PMC9061144 35044839

[7] McDonald AM Lindeman B Bahl D. Radioactive iodine: recognizing the need for risk-benefit balance. J Clin Oncol (2022) 40(13):1396–9. doi: 10.1200/JCO.22.00013 35298297

[8] Leboulleux S Borget I Schlumberger M. Post-operative radioactive iodine administration in patients with low-risk thyroid cancer. Nat Rev Endocrinol (2022) 18(10):585–6. doi: 10.1038/s41574-022-00709-z 35725924

[9] Haugen BR Alexander EK Bible KC Doherty GM Mandel SJ Nikiforov YE. 2015 American thyroid association management guidelines for adult patients with thyroid nodules and differentiated thyroid cancer: the american thyroid association guidelines task force on thyroid nodules and differentiated thyroid cancer. Thyroid (2016) 26(1):1–133. doi: 10.1089/thy.2015.0020 26462967 PMC4739132

[10] Mathew G Agha R Albrecht J Goel P Mukherjee I Pai P. STROCSS 2021: Strengthening the reporting of cohort, cross-sectional and case-control studies in surgery. Int J Surg (2021) 96:106165. doi: 10.1016/j.ijsu.2021.106165 34774726

[11] Sung H Ferlay J Siegel RL Laversanne M Soerjomataram I Jemal A. Global cancer statistics 2020: GLOBOCAN estimates of incidence and mortality worldwide for 36 cancers in 185 countries. CA Cancer J Clin (2021) 71(3):209–49. doi: 10.3322/caac.21660 33538338

[12] Francis GL Waguespack SG Bauer AJ Angelos P Benvenga S Cerutti JM. Management guidelines for children with thyroid nodules and differentiated thyroid cancer. Thyroid (2015) 25(7):716–59. doi: 10.1089/thy.2014.0460 PMC4854274 25900731

[13] Lee IA Kim K Kim JK Kang SW Lee J Jeong JJ. Comparison of surgical outcomes between robotic transaxillary and conventional open thyroidectomy in pediatric thyroid cancer. Cancers (Basel) (2021) 13(13). doi: 10.3390/cancers13133293 PMC8269192 34209221

[14] Spinelli C Piccolotti I Bertocchini A Morganti R Materazzi G Tonacchera M. Familial non-medullary thyroid carcinoma in pediatric age: our surgical experience. World J Surg (2021) 45(8):2473–9. doi: 10.1007/s00268-021-06104-5 PMC8236051 33891138

[15] Hogan AR Zhuge Y Perez EA Koniaris LG Lew JI Sola JE. Pediatric thyroid carcinoma: incidence and outcomes in 1753 patients. J Surg Res (2009) 156(1):167–72. doi: 10.1016/j.jss.2009.03.098 19631341

[16] Chen C Hang L Wu Y Zhang Q Zhang Y Yang J. Retrospective analysis of clinical characteristics and risk factors of differentiated thyroid cancer in children. Front Pediatr (2022) 10:925538. doi: 10.3389/fped.2022.925538 36186657 PMC9516328

[17] Huang D Zhi J Zhang J Qin X Zhao J Zheng X. Relationship between thyroid autoantibodies and recurrence of papillary thyroid carcinoma in children and adolescents. Front Oncol (2022) 12:883591. doi: 10.3389/fonc.2022.883591 35756669 PMC9213685

[18] Gui Y Huang D Hou Y Wei X Zhang J Wang J. Predictive factors for recurrence of papillary thyroid carcinoma in children and adolescents. Front Oncol (2022) 12:833775. doi: 10.3389/fonc.2022.833775 35280803 PMC8909140

[19] Wang X Wang XL. Prognostic analysis of recurrence in children and adolescents with differentiated thyroid cancer. Chin Med J (Engl) (2020) 133(19):2281–6. doi: 10.1097/CM9.0000000000000910 PMC7546846 32941235

[20] Guo K Qian K Shi Y Sun T Chen L Mei D. Clinical and molecular characterizations of papillary thyroid cancer in children and young adults: A multicenter retrospective study. Thyroid (2021) 31(11):1693–706. doi: 10.1089/thy.2021.0003 34514877

[21] Drozd V Saenko V Branovan DI Brown K Yamashita S. Reiners C. A search for causes of rising incidence of differentiated thyroid cancer in children and adolescents after chernobyl and fukushima: comparison of the clinical features and their relevance for treatment and prognosis. Int J Environ Res Public Health (2021) 18(7):3444. doi: 10.3390/ijerph18073444 33810323 PMC8037740

[22] Lee YA Lee H Im SW Song YS Oh DY Kang HJ. NTRK and RET fusion-directed therapy in pediatric thyroid cancer yields a tumor response and radioiodine uptake. J Clin Invest (2021) 131(18). doi: 10.1172/JCI144847 PMC8439610 34237031

[23] Xu Y Wang Y Zhang X Huang R Tian R Liu B. Prognostic value of lymph node ratio in children and adolescents with papillary thyroid cancer. Clin Endocrinol (Oxf) (2021) 95(4):649–56. doi: 10.1111/cen.14491 33914928

[24] Liu Y Wang S Li Y Zhang X Liu Z Liu Q. Clinical Heterogeneity of Differentiated Thyroid Cancer between Children Less than 10 Years of Age and Those Older than 10 Years: A Retrospective Study of 70 Cases. Eur Thyroid J (2021) 10(5):364–71. doi: 10.1159/000516830 PMC8406248 34540706

[25] Zhang X Jiang L Liu L Liu B. Influence of body mass index at diagnosis on outcome of thyroid cancer in children and adolescents. Surgery (2021) 169(6):1373–8. doi: 10.1016/j.surg.2020.12.047 33612290

[26] Wu SS Joshi N Sharrett J Rao S Shah A Scharpf J. Risk factors associated with recurrence and death in patients with tall cell papillary thyroid cancer: A single-institution cohort study with predictive nomogram. JAMA Otolaryngol Head Neck Surg (2023) 149(1):79–86. doi: 10.1001/jamaoto.2022.3781 36454559 PMC9716436

[27] Zanella AB Scheffel RS Weinert L Dora JM Maia AL. New insights into the management of differentiated thyroid carcinoma in children and adolescents (Review). Int J Oncol (2021) 58(5). doi: 10.3892/ijo.2021.5193 33649842

[28] Satapathy S Majeed AK Ballal S Bal C. Differentiated thyroid cancers in young adults versus children: Clinical characteristics and ten-year follow-up outcomes. J Clin Endocrinol Metab (2023) 108(12):e1670–7. doi: 10.1210/clinem/dgad343 37285485

[29] Haddad RI Bischoff L Ball D Bernet V Blomain E Busaidy NL. Thyroid carcinoma, version 2.2022, NCCN clinical practice guidelines in oncology. J Natl Compr Canc Netw (2022) 20(8):925–51. doi: 10.6004/jnccn.2022.0040 35948029

[30] Thomas JK Kurian JJ Cherian AJ Hephzibah J Paul MJ Abraham DT. Papillary thyroid carcinoma in children: clinicopathological profile and outcomes of management. World J Surg (2021) 45(2):496–506. doi: 10.1007/s00268-020-05817-3 33078217

[31] Xu S Huang H Huang Y Qian J Wang X Xu Z. Comparison of lobectomy vs total thyroidectomy for intermediate-risk papillary thyroid carcinoma with lymph node metastasis. JAMA Surg (2023) 158(1):73–9. doi: 10.1001/jamasurg.2022.5781 PMC9713681 36449303

