# Supplementary figures and images for: A novel risk stratification model based on tumor size and multifocality to predict recurrence in pediatric PTC: comparison with adult PTC
Source: Front Endocrinol (Lausanne). 2024 Jan 11;14:1298036. doi: 10.3389/fendo.2023.1298036 (PMC10808709; doi:10.3389/fendo.2023.1298036)

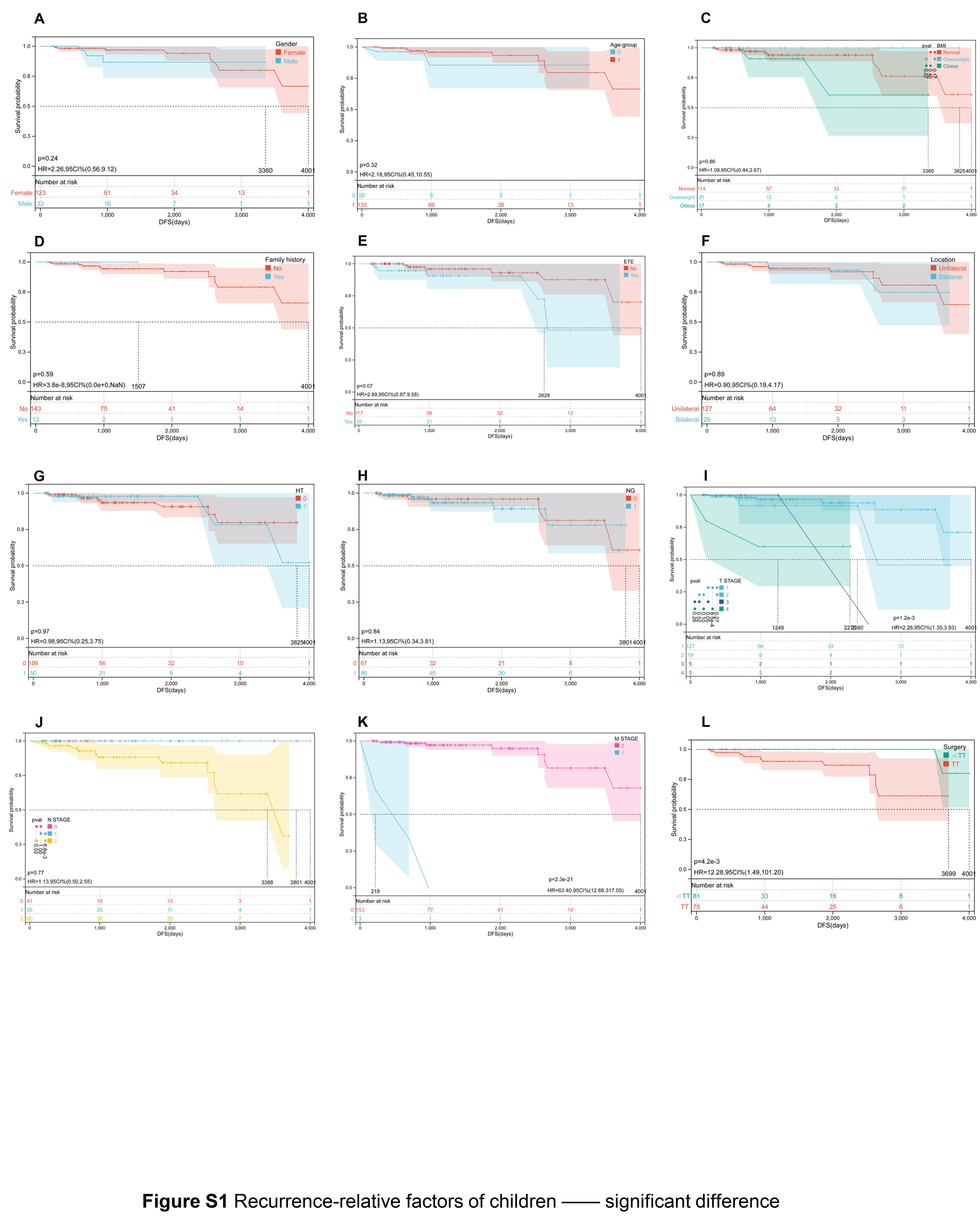

Supplement: Supplementary Figure 1 — Recurrence-relative factors of children. Kaplan−Meier plots of the following: (A) Gender, (B) Age group, (C) BMI, (D) Family history, (E) ETE, (F) Location, (G) HT, (H) NG, (I) T stage, (J) N stage, (K) M stage, and (L) Surgery in pediatric PTC. [file Image_1.tif]
