# Supplementary material for: A novel risk stratification model based on tumor size and multifocality to predict recurrence in pediatric PTC: comparison with adult PTC
Source: Front Endocrinol (Lausanne). 2024 Jan 11;14:1298036. doi: 10.3389/fendo.2023.1298036 (PMC10808709; doi:10.3389/fendo.2023.1298036)
Supplement: Supplementary file 2 [file Table_1.docx]

Table S1 Baseline characteristics of papillary thyroid cancer in adult (N=1297)

| **Characteristics** | **Adult N (%)** | **Characteristics** | **Adult N (%)** |
| --- | --- | --- | --- |
| **Number** | 1244 | Yes | 288(23.15) |
| **Sex** |  | **NG（include）** |  |
| Female | 1030(82.80) | No | 294(23.63) |
| Male | 214(17.20) | Yes | 950(76.37) |
| **Age** | 41.11±9.25 | **T stage** |  |
| **BMI** |  | T1 | 1158(93.09) |
| Normal | 745(61.12) | T2 | 51(4.10) |
| Over-weight | 397(32.57) | T3 | 16(1.29) |
| Obese | 77(6.32) | T4 | 19(1.53) |
| **Tumor size** |  | **N stage** |  |
| ≤1cm | 949(76.47) | N0 | 649(52.17) |
| ＞1cm | 292(23.53) | N1a | 348(27.97) |
| **Extrathyroidal extension** |  | N1b | 247(19.86) |
| No | 881(70.82) | **M stage** |  |
| Yes | 363(29.18) | M0 | 1243(99.92) |
| **Multifocality** |  | M1 | 1(0.08) |
| No | 737(59.24) | **Outcome** |  |
| Yes | 507(40.76) | Non-recurrence | 1212(97.43) |
| **Location** |  | Recurrence | 32(2.57) |
| Unilateral | 890(71.54) | **RAI** |  |
| Bilateral | 354(28.46) | No | 863(69.37) |
| **CLNM** |  | Yes | 337(27.09) |
| No | 696(55.95) | Recommended | 44(3.54) |
| Yes | 548(44.05) | **RRS** |  |
| **LLNM** |  | Low | 688(55.31) |
| No | 254(50.80) | Intermediate | 507(40.76) |
| Yes | 246(49.20) | High | 49(3.94) |
| **HT** |  | **Follow time** (week) | 258.86[57.29,606.71] |
| No | 956(76.85) |  |  |

Abbreviations:BMI,Body Mass Index; ETE, Extrathyroidal extension; HT,Hashimoto's thyroiditis;NG(include),Nodular Goiter;CND,Central Cervical Lymph Node Dissection; LND,Lateral cervical lymph node dissection; CLNM,Central cervical lymph node metastasis; LLNM, Lateral cervical lymph node metastasis; LLNM.cat, Lateral cervical lymph node metastasis-categorical variable;RAI,Radioactive iodine;RRS,Recurrence risk stratification.*P<0.05,**P<0.01,***P<0.001
