# Supplementary material for: A novel risk stratification model based on tumor size and multifocality to predict recurrence in pediatric PTC: comparison with adult PTC
Source: Front Endocrinol (Lausanne). 2024 Jan 11;14:1298036. doi: 10.3389/fendo.2023.1298036 (PMC10808709; doi:10.3389/fendo.2023.1298036)
Supplement: Supplementary file 3 [file Table_2.docx]

Table S2 Clinicopathological characteristics of children papillary thyroid cancer in different age group

| **Characteristics** | **≤14 years N (%)** | **>14 years N (%)** | **Z/χ2** | ***P* value** | **Characteristics** | **≤14 years N (%)** | **>14 years N (%)** | **Z/χ2** | ***P* value** |
| --- | --- | --- | --- | --- | --- | --- | --- | --- | --- |
| **Total** | 26 | 130 |  |  | **NG(include)** |  |  | 4.4 | 0.036* |
| **Sex** |  |  | 0.069 | 0.793 | No | 16(61.5) | 51(39.2) |  |  |
| Female | 21(80.8) | 102(78.5) |  |  | Yes | 10(38.5) | 79(60.8) |  |  |
| Male | 5(19.2) | 28(21.5) |  |  | **T stage** |  |  | -2.956 | 0.003** |
| **Family history** |  |  |  | 0.149 | T1 | 16(61.5) | 111(85.4) |  |  |
| No | 22(84.6) | 121(93.1) | Fisher |  | T2 | 6(23.1) | 13(10.0) |  |  |
| Yes | 4(15.4) | 9(6.9) |  |  | T3 | 0 (0) | 5 (3.8) |  |  |
| **Tumor size** |  |  | 5.932 | 0.015* | T4 | 4 (15.4) | 1 (0.8) |  |  |
| ≤1cm | 7(26.9) | 69(53.1) |  |  | **N stage** |  |  | -2.923 | 0.003** |
| >1cm | 19(73.1) | 61(46.9) |  |  | N0 | 2(7.7) | 39(30) |  |  |
| **ETE** |  |  | 0.554 | 0.457 | N1a | 8 (30.8) | 47(36.2) |  |  |
| No | 18(69.2) | 99(76.2) |  |  | N1b | 16(61.5) | 44(33.8) |  |  |
| Yes | 8(30.8) | 31(23.8) |  |  | **Treatment** |  |  | 0.416 | 0.519 |
| **Multifocality** |  |  | 1.311 | 0.252 | <TT | 12(46.2) | 69(53.1) |  |  |
| No | 15(57.7) | 90(69.2) |  |  | TT | 14(53.8) | 61 (46.9) |  |  |
| Yes | 11(42.3) | 40(30.8) |  |  | **Outcome** |  |  |  | 0.577 |
| **Location** |  |  |  | 0.557 | Non-recurrence | 24(92.3) | 121(93.1) | Fisher |  |
| Unilateral | 21(80.8) | 106(81.5) | Fisher |  | Recurrence | 2 (7.7) | 9(6.9) |  |  |
| Bilateral | 5(19.2) | 24(18.5) |  |  | **RAI** |  |  | 0.633 | 0.426 |
| **CLNM** |  |  | 6.483 | 0.011* | No | 13(50.0) | 76(58.5) |  |  |
| No | 2(7.7) | 42(32.3) |  |  | Yes | 13(50.0) | 54(41.5) |  |  |
| Yes | 24(92.3) | 88(67.7) |  |  | **Complications** |  |  | Fisher | 0.088 |
| **LLNM** |  |  |  | 0.048* | No | 22(84.6) | 123(94.6) |  |  |
| No | 0(0) | 12(21.8) | Fisher |  | Yes | 4 (15.4) | 7 (5.4) |  |  |
| Unilateral | 11(68.8) | 35(63.6) |  |  | **RRS** |  |  | -1.68 | 0.093 |
| Bilateral | 5(31.3) | 8 (14.5) |  |  | Low | 2(7.7) | 41(31.5) |  |  |
| **HT** |  |  | 6.805 | 0.009** | Intermediate | 14(53.8) | 46(35.4) |  |  |
| No | 12(46.2) | 94(72.3) |  |  | High | 10 (38.5) | 43(33.1) |  |  |
| Yes | 14(53.8) | 36(27.7) |  |  |  |  |  |  |  |

Abbreviations:BMI,Body Mass Index; ETE, Extrathyroidal extension; HT,Hashimoto's thyroiditis;NG(include),Nodular Goiter;CND,Central Cervical Lymph Node Dissection; LND,Lateral cervical lymph node dissection; CLNM,Central cervical lymph node metastasis; LLNM, Lateral cervical lymph node metastasis; LLNM.cat, Lateral cervical lymph node metastasis-categorical variable;RAI,Radioactive iodine;RRS,Recurrence risk stratification.*P<0.05,**P<0.01,***P<0.001
