# Supplementary material for: A novel risk stratification model based on tumor size and multifocality to predict recurrence in pediatric PTC: comparison with adult PTC
Source: Front Endocrinol (Lausanne). 2024 Jan 11;14:1298036. doi: 10.3389/fendo.2023.1298036 (PMC10808709; doi:10.3389/fendo.2023.1298036)
Supplement: Supplementary file 4 [file Table_3.docx]

Table S3 Relevant factors of central lymph node metastasis in children with papillary thyroid cancer

| **Characteristics** | **without N (%)** | **with N (%)** | **Z/χ2** | ***P* value** | **Characteristics** | **without N (%)** | **with N (%)** | **Z/χ2** | ***P* value** |
| --- | --- | --- | --- | --- | --- | --- | --- | --- | --- |
| **Total** | 44 | 112 |  |  | **NG(include)** |  |  | 0.157 | 0.692 |
| **Age** |  |  | 6.483 | 0.011* | No | 20(45.5) | 47(42.0) |  |  |
| ≤14 | 42(95.5) | 88(78.6) |  |  | Yes | 24(54.5) | 65(58.0) |  |  |
| ＞14 | 2(4.5) | 24(21.4) |  |  | **T stage** |  |  | -3.286 | 0.001** |
| **Sex** |  |  | 2.077 | 0.15 | T1 | 43(97.7) | 84(75.0) |  |  |
| Female | 38(86.4) | 85(75.9) |  |  | T2 | 1 (2.3) | 18(16.1) |  |  |
| Male | 6(13.6) | 27(24.1) |  |  | T3 | 0 (0) | 5(4.5) |  |  |
| **Family history** |  |  |  | 0.354 | T4 | 0 (0) | 5(4.5) |  |  |
| No | 42(95.5) | 101(90.2) | Fisher |  | **N stage** |  |  | -9.287 | < 0.001*** |
| Yes | 2(4.5) | 11 (9.8) |  |  | N0 | 41(93.2) | 0 (0) |  |  |
| **Tumor size** |  |  | 23.31 | < 0.001*** | N1a | 0 (0) | 55(49.1) |  |  |
| ≤1cm | 35(79.5) | 41(36.6) |  |  | N1b | 3 (6.8) | 57(50.9) |  |  |
| >1cm | 9(20.5) | 71(63.4) |  |  | **Treatment** | |  | 25.404 | < 0.001*** |
| **ETE** |  |  | 0.675 | 0.411 | <TT | 37(84.1) | 44(39.3) |  |  |
| No | 35(79.5) | 82(73.2) |  |  | TT | 7 (15.9) | 68(60.7) |  |  |
| Yes | 9(20.5) | 30(26.8) |  |  | **Outcome** |  |  |  | 0.023* |
| **Multifocality** |  |  | 0.818 | 0.366 | Recurrence | 0 (0) | 11 (9.8) | Fisher |  |
| No | 32(72.7) | 73(65.2) |  |  | Non-recurrence | 44 (100) | 101(90.2) |  |  |
| Yes | 12(27.3) | 39(34.8) |  |  | **RAI** |  |  | 28.672 | < 0.001*** |
| **Location** |  |  | 2.115 | 0.146 | No | 40(90.9) | 49(43.8) |  |  |
| Unilateral | 39(88.6) | 88(78.6) |  |  | Yes | 4(9.1) | 63(56.3) |  |  |
| Bilateral | 5 (11.4) | 24(21.4) |  |  | **Complications** |  |  |  | 0.13 |
| **LLNM** |  |  |  | 0.001* | No | 43(97.7) | 102(91.1) | Fisher |  |
| No | 6(66.7) | 6(9.7) | Fisher |  | Yes | 1(2.3) | 10 (8.9) |  |  |
| Unilateral | 3 (33.3) | 43(69.4) |  |  | **RRS** |  |  | 114.561 | < 0.001*** |
| Bilateral | 0 (0) | 13(21.0) |  |  | Low | 39(88.6) | 4(3.6) |  |  |
| **HT** |  |  | 0.002 | 0.969 | Intermediate | 2(4.5) | 58(51.8) |  |  |
| No | 30(68.2) | 76(67.9) |  |  | High | 3 (6.8) | 50(44.6) |  |  |
| Yes | 14(31.8) | 36(32.1) |  |  |  |  |  |  |  |

Abbreviations:BMI,Body Mass Index; ETE, Extrathyroidal extension; HT,Hashimoto's thyroiditis;NG(include),Nodular Goiter;CND,Central Cervical Lymph Node Dissection; LND,Lateral cervical lymph node dissection; CLNM,Central cervical lymph node metastasis; LLNM, Lateral cervical lymph node metastasis; LLNM.cat, Lateral cervical lymph node metastasis-categorical variable;RAI,Radioactive iodine;RRS,Recurrence risk stratification.*P<0.05,**P<0.01,***P<0.001
