# Supplementary material for: A novel risk stratification model based on tumor size and multifocality to predict recurrence in pediatric PTC: comparison with adult PTC
Source: Front Endocrinol (Lausanne). 2024 Jan 11;14:1298036. doi: 10.3389/fendo.2023.1298036 (PMC10808709; doi:10.3389/fendo.2023.1298036)
Supplement: Supplementary file 5 [file Table_4.docx]

Table S4 Relevant factors of lateral neck lymph node metastasis in children with papillary thyroid cancer

| **Characteristics** | **without N (%)** | **with N (%)** | **Z/χ2** | ***P* value** | **Characteristics** | **without N (%)** | **with N (%)** | **Z/χ2** | ***P* value** |
| --- | --- | --- | --- | --- | --- | --- | --- | --- | --- |
| **Total** | 12 | 59 |  |  | **NG（include）** |  |  | 0.142 | 0.707 |
| **Age** |  |  |  | 0.034* | No | 6(50) | 26(44.1) |  |  |
| ≤14 | 0(0) | 16(27.1) | Fisher |  | Yes | 6(50) | 33(55.9) |  |  |
| ＞14 | 12(100) | 43(72.9) |  |  | **T stage** |  |  | -2.641 | 0.008 |
| **Sex** |  |  |  | 0.044* | T1 | 12(100) | 35(59.3) |  |  |
| Female | 12(100) | 44(74.6) | Fisher |  | T2 | 0 (0) | 15(25.4) |  |  |
| Male | 0(0) | 15(25.4) |  |  | T3 | 0 (0) | 4 (6.8) |  |  |
| **Family history** |  |  |  | 0.831 | T4 | 0 (0) | 5 (8.5) |  |  |
| No | 12(100) | 58(98.3) | Fisher |  | **N stage** |  |  | -8.331 | < 0.001*** |
| Yes | 0(0) | 1(1.7) |  |  | N0 | 6(50) | 0 (0) |  |  |
| **Tumor size** | |  |  | < 0.001*** | N1a | 6(50) | 0 (0) |  |  |
| ≤1cm | 10(83.3) | 12(20.3) | Fisher |  | N1b | 0 (0) | 59(100) |  |  |
| >1cm | 2(16.7) | 47(79.7) |  |  | **Treatment** | |  |  | 0.002 |
| **ETE** |  |  |  | 0.125 | <TT | 8(66.7) | 11(18.6) | Fisher |  |
| No | 10(83.3) | 36(61.0) | Fisher |  | TT | 4(33.3) | 48(81.4) |  |  |
| Yes | 2(16.7) | 23(39.0) |  |  | **Outcome** |  |  |  | 0.136 |
| **Multifocality** | |  | 3.875 | 0.049* | Recurrence | 12(100) | 49(83.1) | Fisher |  |
| No | 10(83.3) | 31(52.5) |  |  | Non-recurrence | 0 (0) | 10(16.9) |  |  |
| Yes | 2(16.7) | 28(47.5) |  |  | **RAI** |  |  |  | 0.005 |
| **Location** |  |  |  | 0.087 | No | 7(58.3) | 10(16.9) | Fisher |  |
| Unilateral | 11(91.7) | 40(67.8) | Fisher |  | Yes | 5(41.7) | 49(83.1) |  |  |
| Bilateral | 1(8.3) | 19(32.2) |  |  | **Complications** |  |  |  | 0.593 |
| **CLNM** |  |  |  | < 0.001*** | No | 11(91.7) | 52(88.1) | Fisher |  |
| No | 6(50) | 3 (5.1) | Fisher |  | Yes | 1(8.3) | 7 (11.9) |  |  |
| Yes | 6(50) | 56 (94.9) |  |  | **RRS** |  |  | -6.777 | < 0.001*** |
| **HT** |  |  |  | 0.547 | Low | 6(50) | 0 (0) |  |  |
| No | 9(75) | 42(71.2) | Fisher |  | Intermediate | 6(50) | 6(10.2) |  |  |
| Yes | 3(25) | 17(28.8) |  |  | High | 0 (0) | 53(89.8) |  |  |

Abbreviations:BMI,Body Mass Index; ETE, Extrathyroidal extension; HT,Hashimoto's thyroiditis;NG(include),Nodular Goiter;CND,Central Cervical Lymph Node Dissection; LND,Lateral cervical lymph node dissection; CLNM,Central cervical lymph node metastasis; LLNM, Lateral cervical lymph node metastasis; LLNM.cat, Lateral cervical lymph node metastasis-categorical variable;RAI,Radioactive iodine;RRS,Recurrence risk stratification.*P<0.05,**P<0.01,***P<0.001
